# Prions and Prion Diseases: Current Perspectives

**DOI:** 10.3201/eid1012.040847

**Published:** 2004-12

**Authors:** Ermias D. Belay

**Affiliations:** * Centers for Disease Control and Prevention, Atlanta, Georgia, USA

**Keywords:** Prions and Prion Diseases: Current Perspectives, Glenn C. Telling

Prion diseases, also known as transmissible spongiform encephalopathies, are rapidly progressive, uniformly fatal brain diseases that can infect humans and animals, including cattle, sheep, goats, mink, deer, elk, cats, and zoo ungulates. In humans, prion diseases can occur as a sporadic or inherited disease, or as a result of iatrogenic transmission. Prion diseases generated great public concern after an outbreak of bovine spongiform encephalopathy occurred in many European countries and scientific evidence indicated its transmission to humans.

Research in prion diseases is hampered by certain unconventional properties of the presumed etiologic agent and the long incubation period associated with these diseases. Most conventional laboratory methods used to study viruses and bacteria may not be applicable. In the past, the etiologic agent of transmissible spongiform encephalopathies was believed to be a slow virus, primarily because of its transmissibility, ability to retain infectivity after filtration, and long incubation period. The successful transmission of scrapie, a centuries-old prion disease of sheep, to mice in 1961 greatly facilitated identification and characterization of the scrapie agent. Several characteristics of the scrapie agent suggest that the agent is not a virus but is likely composed primarily of a protein. The agent's characteristics include the absence of disease-specific nucleic acids; resistance to radiation, nucleases, and standard sterilization and disinfection methods; and inactivation by protein-modifying procedures. These observations and purification of the scrapie prion in the early 1980s led to widespread acceptance of the prion hypothesis.

Since the 1980s, both the scope and nature of prion disease research has progressed rapidly. The economic and human cost associated with the bovine spongiform encephalopathy outbreak fueled the need to better understand the etiologic agent of prion diseases and their basic transmission mechanism. Prions and Prion Diseases: Current Perspectives summarizes the advances in prion disease research. It expands on a previous volume edited by David Harris that was published in 1999 under the title Prions: Molecular and Cellular Biology. The book's 10 chapters describe the biochemical and molecular features of prions and the normal prion protein, various laboratory methods for studying prions, and advances in the pathogenesis and immunology of prion diseases.

Chapters 2 through 6 detail laboratory methods developed to study the unconventional agent of prion diseases. Chapter 2 describes a cell-free conversion reaction system to study how pathogenic prions associated with different species interact with host cellular prion protein. Such systems have been used to study the biochemical mechanisms of prion diseases and can potentially be used to screen new therapies for their effectiveness against prion diseases. Chapter 3 describes the mechanisms underlying the biosynthesis and cell biology of the cellular prion protein by using cell culture systems. Understanding the detailed biochemical properties of the cellular prion protein will help show the molecular basis of its interaction with, and conversion to, the pathogenic prions. Subsequent chapters in the book describe other laboratory methods, including transgenic mouse models, which can be used to investigate the transmissibility of prions among different species, the extent and degree of the "species barrier," the mechanism of prion propagation, and prion disease pathogenesis.

Overall, the book provides a wealth of information on the progress made in understanding the molecular, immunologic, and genetic aspects of prion diseases and the laboratory methods used to study them. This book will be valuable to prion disease researchers, to scientists who want to gain more knowledge about the progress made in understanding the mechanisms of prion propagation, and to persons just beginning to study these unconventional, fatal brain diseases.

